# Reversible choroidal ischemia as a rare sight-threatening manifestation of microscopic polyangiitis presenting with crescentic glomerulonephritis

**DOI:** 10.1186/s12882-026-04828-x

**Published:** 2026-04-02

**Authors:** Gamze İçaçan, Neslihan Güney, Faruk Bıçak

**Affiliations:** 1 Department of Nephrology, University of Health Sciences, İzmir Faculty of Medicine, İzmir, Turkey; 2 Department of Pathology, University of Health Sciences, İzmir Faculty of Medicine, İzmir, Turkey; 3 Department of Ophthalmology, University of Health Sciences, İzmir Faculty of Medicine, İzmir, Turkey

**Keywords:** Microscopic polyangiitis, ANCA-associated vasculitis, Crescentic glomerulonephritis, Choroidal ischemia, Fluorescein angiography

## Abstract

**Background:**

Ocular involvement in ANCA-associated vasculitis is uncommon and usually affects the anterior segment. Posterior segment ischemic involvement, particularly choroidal perfusion disturbance, is extremely rare. We report a patient with microscopic polyangiitis (MPA) who developed bilateral visual impairment due to choroidal perfusion impairment demonstrated by fluorescein angiography (FFA), along with severe crescentic glomerulonephritis.

**Case presentation:**

A 53-year-old woman presented with acute kidney injury, proteinuria, hematuria, elevated inflammatory markers, and p-ANCA positivity. Kidney biopsy demonstrated pauci-immune crescentic glomerulonephritis. She was treated with pulse steroids, cyclophosphamide, and plasma exchange. Ten days later, she developed bilateral visual loss. Fundus examination revealed preserved retinal vasculature without hemorrhage or exudates. FFA showed normal retinal vessel filling without vascular leakage but diffuse patchy background hypofluorescence, predominantly at the posterior pole, consistent with choroidal perfusion impairment. Immunosuppression was continued, leading to complete visual recovery and partial renal improvement.

**Conclusion:**

Recognition of this entity and prompt immunosuppressive treatment may prevent irreversible vision loss.

**Supplementary Information:**

The online version contains supplementary material available at 10.1186/s12882-026-04828-x.

## Learning Points

Renal involvement is common in ANCA-associated vasculitis, whereas choroidal ischemia is rare.

Visual loss in AAV is a serious complication that requires urgent diagnosis and treatment.

The coexistence of renal and ocular involvement reflects the multisystemic nature of the disease.

Early immunosuppression and plasma exchange may positively influence both renal and visual prognosis.

**Supplementary Information:**

The online version contains supplementary material available at 10.1186/s12882-026-04828-x.

## Introduction

Antineutrophil cytoplasmic antibody (ANCA)-associated vasculitides (AAV) are a group of rare, immune-mediated systemic diseases affecting small vessels. According to the 2013 Chapel Hill Consensus Conference classification, AAV includes three main subtypes: granulomatosis with polyangiitis (GPA), microscopic polyangiitis (MPA), and eosinophilic granulomatosis with polyangiitis (EGPA) [1]. The most important characteristic of AAV is its systemic course and the potential to cause severe, life-threatening involvement of multiple organs.

Renal involvement is the most common and prognostically decisive clinical feature of AAV. In MPA, kidney disease typically presents as pauci-immune necrotizing crescentic glomerulonephritis [2]. This histopathological finding is associated with rapidly progressive renal failure and end-stage kidney disease. In the international cohort study by Tanna et al. [3], renal involvement was shown to be the strongest determinant of both mortality and renal survival. Therefore, kidney biopsy plays a critical diagnostic and prognostic role in MPA [2, 3].

Ocular involvement in AAV is less common; however, when it occurs it may lead to highly significant clinical consequences. In the literature, ocular manifestations are most frequently reported as episcleritis, scleritis, ocular inflammation, or orbital involvement [4]. Posterior segment involvement is extremely rare, and within this group, ischemic impairment of the choroidal circulation has been described only in a very limited number of cases. Due to its dense vascular network, high oxygen demand, and marked metabolic activity, the choroid is particularly vulnerable to injury in immune-mediated vasculitic processes. Involvement of the choroidal vasculature may lead to perfusion impairment and ischemia, resulting in severe visual loss. Therefore, despite its rarity, ischemic choroidal involvement is considered clinically critical [5,6].

Recent studies have demonstrated that ocular involvement in ANCA-associated vasculitis may present with a broader spectrum than previously assumed, and that it is not limited to the anterior segment but may also affect posterior segment structures. However, perfusion impairment secondary to involvement of the choroidal circulation is reported far less frequently in the literature compared with retinal vascular pathology, which may lead to diagnostic challenges. Particularly in systemic vasculitis cases accompanied by severe renal involvement, careful evaluation of ocular findings and recognition of posterior segment involvement are of critical importance [4–6].

Multisystem involvement in MPA is an important clinical feature that reflects the severity and heterogeneity of the disease. Ocular manifestations accompanying severe renal involvement may be considered an indicator of systemic inflammatory activity. Therefore, sudden visual loss developing in AAV patients represents a critical condition requiring urgent evaluation and aggressive treatment.

In this report, we present a rare MPA case who initially presented with severe renal involvement and subsequently developed ischemic ocular involvement consistent with choroidal perfusion impairment during treatment. This case highlights the coexistence of rare ocular involvement and severe renal disease in AAV and emphasizes the importance of a multidisciplinary approach.

## Case presentation

A 53-year-old woman with a known history of hypertension and rheumatoid arthritis presented with complaints of fever, fatigue, and loss of appetite persisting for approximately one month. Physical examination revealed no remarkable pathological findings. On admission, her blood pressure was 140/90 mmHg. Her height was 158 cm, weight was 75 kg, and body mass index (BMI) was 30.0 kg/m². Prior to admission, she was taking valsartan/hydrochlorothiazide (160/12.5 mg/day), methylprednisolone (4 mg/day), and pantoprazole (40 mg/day). At admission, her serum creatinine level was 7.6 mg/dL, and she was hospitalized in the nephrology department with the preliminary diagnosis of acute kidney injury. Laboratory findings on admission are summarized in Table 1. Urinalysis revealed significant hematuria and proteinuria (2+). Twenty-four-hour urine protein excretion was 1.3 g/day. Further investigations demonstrated normal liver enzymes and immunoglobulin levels, with no monoclonality on serum protein electrophoresis and a normal serum free light chain κ/λ ratio (1.23). Autoimmune serology revealed negative ANA and anti-dsDNA results, with normal complement levels (C3 and C4). ANCA testing by indirect immunofluorescence revealed p-ANCA positivity (titre 1:320), and ELISA-based testing showed MPO-ANCA positivity with negative PR3-ANCA, supporting the preliminary diagnosis of ANCA-associated vasculitis. Thoracic high-resolution computed tomography, paranasal sinus computed tomography, and consultations with pulmonology, otorhinolaryngology, and ophthalmology were performed to evaluate extrarenal involvement, with no findings suggestive of vasculitic involvement.

**Table 1:** Laboratory findings on admission (with reference ranges)

Parameter	Result	Unit	Reference range
Blood urea nitrogen (BUN)	57	mg/dL	7–20
Creatinine	7.65	mg/dL	0.6–1.1
Hemoglobin	9.8	g/dL	12.0–16.0
White blood cell count (WBC)	11,410	/µL	4,000–10,000
Platelet count	378,000	/µL	150,000–400,000
Albumin	3.7	g/dL	3.5–5.0
Aspartate aminotransferase (AST)	32	U/L	0–35
Alanine aminotransferase (ALT)	26	U/L	0–45
C-reactive protein (CRP)	126	mg/L	< 5
Procalcitonin	0.34	ng/mL	< 0.05
Erythrocyte sedimentation rate (ESR)	97	mm/h	0–20
Ferritin	249	ng/mL	15–150
Complement C3	0.92	g/L	0.90–1.80
Complement C4	0.20	g/L	0.10–0.40
Antinuclear antibody (ANA)	Negative	–	Negative
Anti–double-stranded DNA (anti-dsDNA)	Negative	–	Negative
p-ANCA (indirect immunofluorescence)	Positive (1:320)	–	Negative
Myeloperoxidase-ANCA (MPO-ANCA)	Positive	–	Negative
Proteinase 3-ANCA (PR3-ANCA)	Negative	–	Negative
Immunoglobulin G (IgG)	10.0	g/L	7.0–16.0
Immunoglobulin A (IgA)	2.6	g/L	0.7–4.0
Immunoglobulin M (IgM)	1.1	g/L	0.4–2.3
Serum free light chain κ/λ ratio	1.23	–	0.26–1.65
Serum protein electrophoresis	No monoclonality detected	–	No monoclonality
Urinalysis (erythrocytes)	353	/HPF	0–2
Urinalysis (protein)	2+	semi-quantitative	Negative/trace
24-h urine protein	1.3	g/day	< 0.15

Footnote: Reference ranges may vary slightly between laboratories

**Fig. 1:**
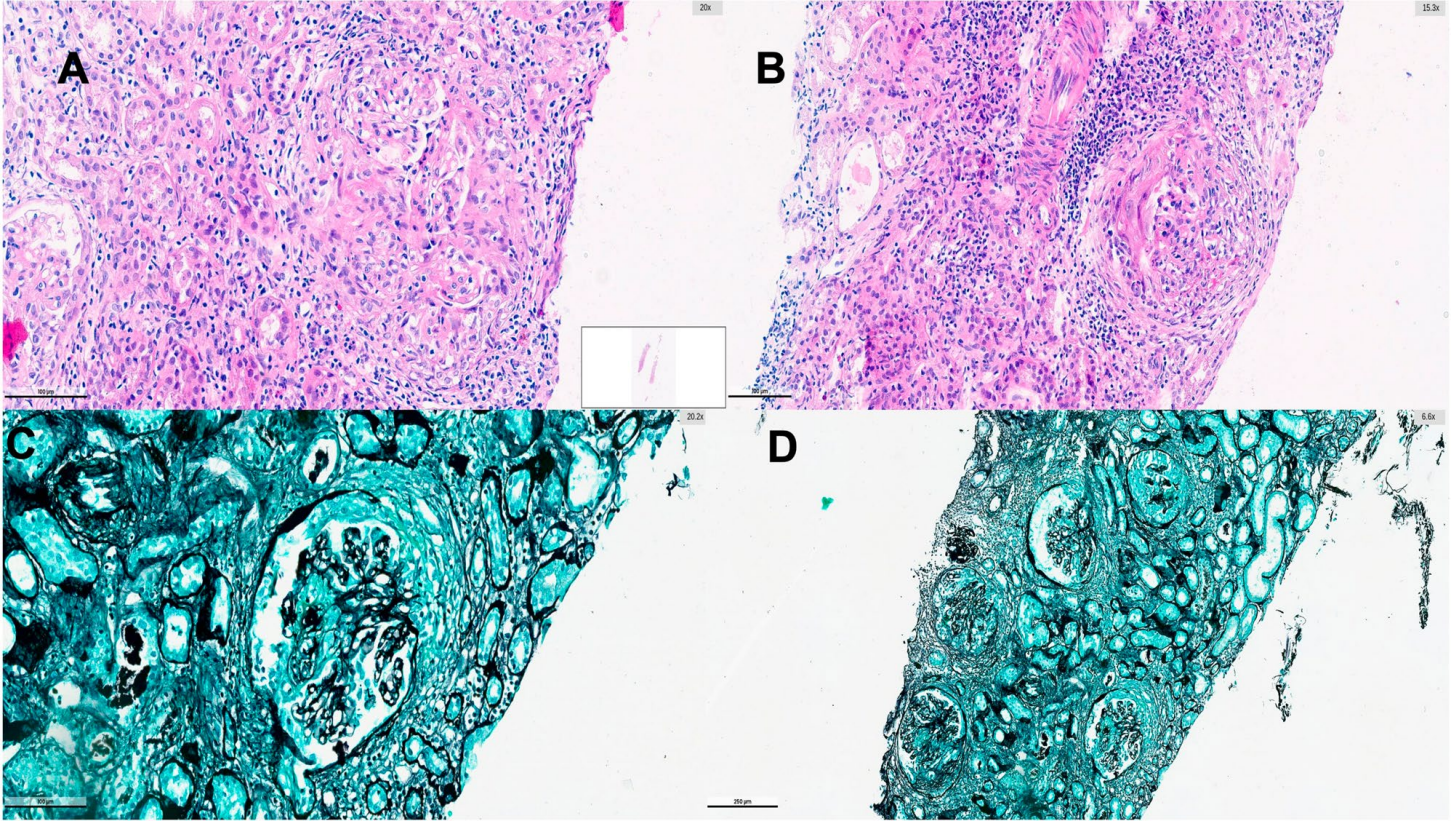
Renal biopsy findings. (**A–B**) Hematoxylin and eosin staining demonstrating necrotizing crescentic glomerulonephritis with prominent cellular crescents, inflammatory infiltration, and fibrinoid necrosis. (**C–D**) Silver staining showing disrupted glomerular basement membrane architecture and crescent formation consistent with crescentic vasculitic nephritis

Renal biopsy revealed prominent crescent formation and fibrinoid necrosis, with immunofluorescence findings consistent with a pauci-immune pattern (Fig. 1). Histopathological findings were reported as pauci-immune necrotizing and crescentic glomerulonephritis, compatible with ANCA-associated vasculitis. In light of these findings, the patient was diagnosed with microscopic polyangiitis. A summary of the renal histopathology report with all patient-identifying information removed is provided in Supplementary Appendix 1.

Pulse intravenous methylprednisolone (1 g/day for 3 days in total) was initiated as induction therapy, followed by oral prednisolone at 1 mg/kg/day. Intravenous cyclophosphamide was administered at a dose of 750 mg at 3-week intervals for a total of six doses. Due to severe renal involvement, hemodialysis requirement, and oliguria, conventional intermittent hemodialysis was initiated, and the patient underwent a total of four sessions during the first 8 days of hospitalization (three times per week schedule). In addition, seven sessions of plasma exchange were performed using a membrane-based technique, with fresh frozen plasma (FFP) as the replacement fluid.

On day 19 of treatment, the patient developed blurred vision and decreased visual acuity. Visual acuity was 2/10 in the right eye and 4/10 in the left eye. Slit-lamp examination showed no evidence of anterior uveitis (no anterior chamber cells/flare) or intermediate uveitis (no vitritis). Fundus examination revealed no retinal hemorrhage, exudate, or signs of retinal vasculitis. Fundus examination suggested subtle blurring of the right optic disc margin; however, fluorescein angiography demonstrated mild optic disc staining without definite leakage, and no definite optic disc edema was observed. Fluorescein angiography confirmed the absence of retinal vasculitis and optic disc leakage. Optical coherence tomography showed no cystoid macular edema but demonstrated irregularities in the outer retinal layers (Fig. 3).

**Fig. 2:**
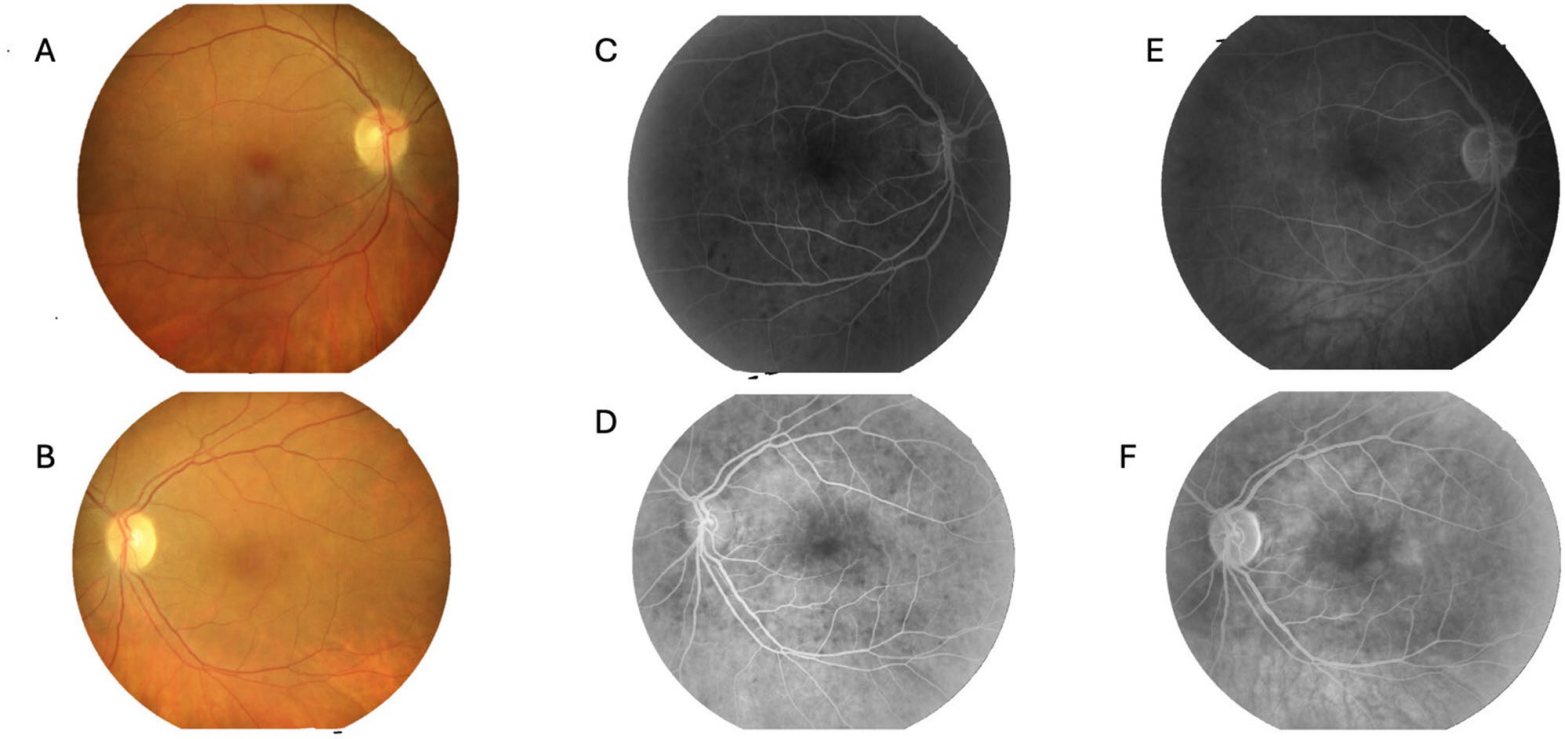
Color fundus photography and fluorescein angiography findings. (**A–B**) Color fundus photographs of the right (OD) and left (OS) eyes. (**C–D**) Early-phase fluorescein angiography images of OD and OS demonstrating delayed and patchy choroidal filling despite preserved retinal vascular filling. (**E–F**) Late-phase fluorescein angiography images of OD and OS showing mild optic disc staining without definite leakage and no evidence of retinal vasculitis

As shown in Fig. 2, retinal vascular filling was preserved; however, diffuse irregular background hypofluorescence, more prominent in the posterior pole, was observed, consistent with impaired choroidal perfusion and ischemia.

**Fig. 3:**
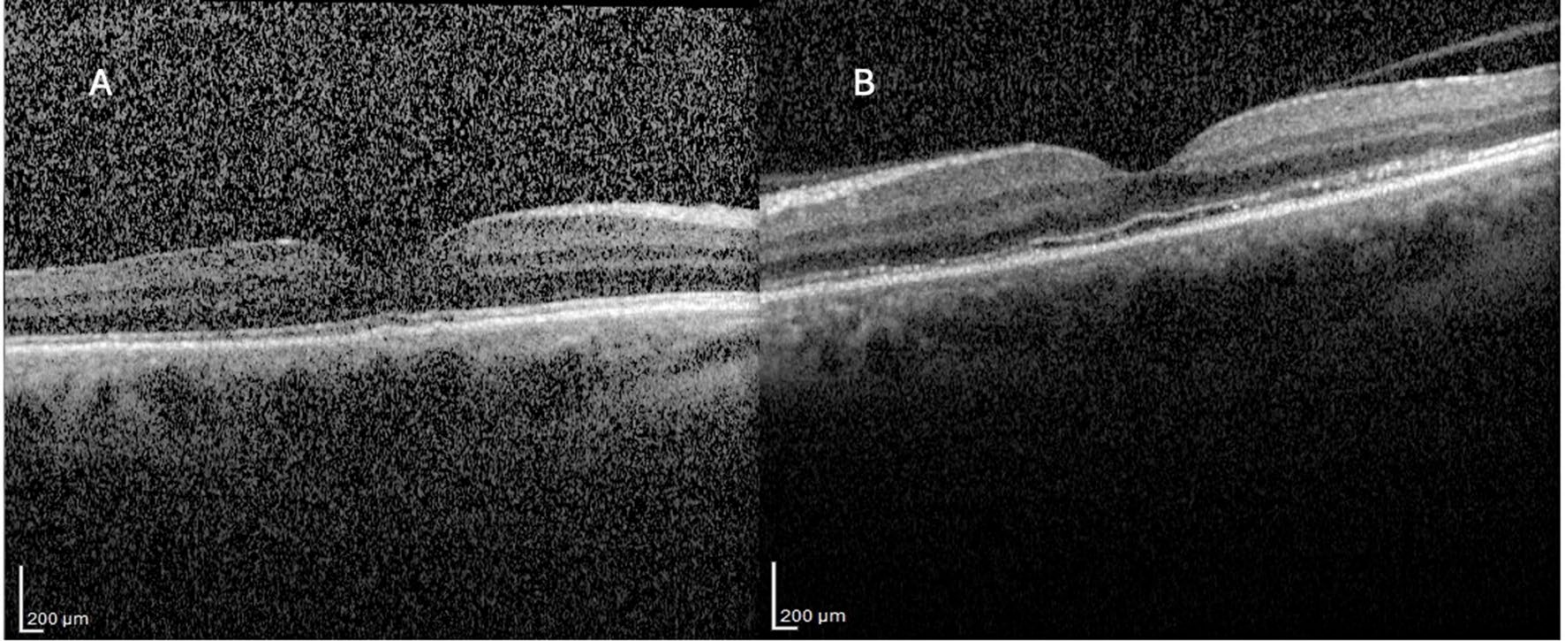
Optical coherence tomography (OCT) findings at the time of visual deterioration. (**A**) Right eye (OD) OCT demonstrating outer retinal layer irregularities without cystoid macular edema. (**B**) Left eye (OS) OCT demonstrating similar outer retinal changes without cystoid macular edema

Following the second dose of cyclophosphamide and continuation of methylprednisolone at 1 mg/kg/day, visual acuity improved to 10/10 in both eyes by the third day. Follow-up fluorescein angiography demonstrated complete resolution of choroidal ischemia. During follow-up, visual function returned entirely to normal, partial improvement was observed in renal function tests, and the patient no longer required hemodialysis.

## Discussion

ANCA-associated vasculitides are systemic diseases characterized by multiorgan involvement and may lead to high morbidity and mortality. Renal involvement is the most common manifestation of AAV and is one of the most important determinants of prognosis. Microscopic polyangiitis (MPA) generally presents with renal involvement and is frequently characterized by pauci-immune necrotizing crescentic glomerulonephritis [1, 2]. In our patient, a serum creatinine level of 7.6 mg/dL at the time of diagnosis and crescent formation in 14 glomeruli on biopsy indicated severe renal involvement. Previous studies have demonstrated that the presence of crescentic GN is strongly associated with early renal function loss and is an important determinant of renal survival [3].

Although ocular involvement in AAV is relatively uncommon, it represents an important cause of morbidity when it occurs. In a study by Mohammad et al. [4], ocular involvement was reported in approximately 10–15% of AAV patients, most commonly in the form of episcleritis, scleritis, or orbital inflammation. Although posterior segment involvement is generally reported as retinal vascular pathology, ischemic impairment of the choroidal circulation is extremely rare and presents significant diagnostic and management challenges. In a study evaluating 14 AAV patients followed for retinal vasculitis between 2006 and 2017, choroidal vasculitis was identified in four cases; however, all of these cases were reported to have GPA.

Due to its dense vascular network, high oxygen consumption, and metabolic activity, the choroid is highly susceptible to immune-inflammatory processes involving the vessel wall. Therefore, impairment of choroidal perfusion in systemic vasculitic processes is considered a critical complication that may result in severe vision loss [4–6].

In the present case, the development of ischemic involvement consistent with choroidal perfusion impairment simultaneously with severe renal disease in the course of MPA is remarkable. Following the sudden onset of visual loss, fundus examination and imaging studies revealed normal retinal vessels, suggesting that the primary pathology was at the choroidal level. In fluorescein angiography, preservation of retinal vascular filling together with diffuse and irregular background hypofluorescence, particularly in the posterior pole, provided important evidence supporting the presence of perfusion loss and ischemia in the choroidal circulation. In this respect, our case is similar to previously reported rare cases of choroidal ischemic involvement associated with MPA in the literature [7].

Although the exact mechanism underlying the development of choroidal ischemia has not been fully clarified, it is thought that immune-mediated endothelial injury at the level of small vessels, vascular wall inflammation, luminal narrowing, and accompanying microthrombotic processes may lead to perfusion loss [8]. It has been suggested that during periods of high systemic inflammatory activity in AAV, particularly in cases accompanied by severe renal involvement, vasculitic processes may be more widespread and may also affect the ocular vascular bed. In our patient, the emergence of choroidal perfusion impairment concurrently with systemic disease activity, and its rapid improvement following intensification of immunosuppressive therapy, support this pathophysiological mechanism [2, 4].

Indocyanine green angiography (ICGA) is considered the gold standard for the evaluation of choroidal circulation. However, ICGA could not be performed in our patient because it was not available in our clinic at the time of evaluation. In this context, fundus fluorescein angiography (FA) findings were carefully assessed. Despite preserved retinal vascular filling in both eyes, early-phase FA demonstrated delayed and patchy choroidal filling, which was considered compatible with choroidal ischemia. In our case, OCT did not demonstrate cystoid macular edema but showed irregularities in the outer retinal layers, which may reflect secondary involvement of the outer retina and retinal pigment epithelium due to impaired choroidal perfusion (Fig. 3). Previous reports have indicated that, particularly in vasculitis-related choroidal ischemia, early-phase choroidal filling delay on FA in the presence of preserved retinal circulation may serve as a diagnostically meaningful indicator when ICGA is unavailable [9].

Choroidal ischemia can also occur secondary to non-vasculitic causes, particularly systemic hemodynamic disturbances such as acute hypotension, marked blood pressure fluctuations, severe anemia, or rapid intravascular volume shifts. Initiation of hemodialysis may theoretically precipitate transient ocular hypoperfusion in susceptible patients, most commonly in the setting of intradialytic hypotension. However, in our case, no episodes of intradialytic hypotension were documented, and the patient remained volume overloaded due to oliguria, making dialysis-related hypotension a less likely primary trigger. Taken together with MPO-ANCA positivity and biopsy-proven ANCA-associated crescentic glomerulonephritis, the clinical and angiographic findings are more consistent with vasculitic choroidal involvement. Nevertheless, we acknowledge that hemodynamic factors may contribute in selected cases, and careful monitoring of blood pressure and volume status is warran.

In terms of treatment, the gold-standard induction therapy in AAV consists of high-dose corticosteroids in combination with cyclophosphamide or rituximab. The KDIGO 2024 Clinical Practice Guideline for the Management of Antineutrophil Cytoplasmic Antibody (ANCA)–Associated Vasculitis recommends plasma exchange in selected patients with severe renal involvement [10]. In our case, a combination of high-dose steroids, cyclophosphamide, and plasma exchange was administered, resulting in significant improvement in renal function (creatinine 7.6 → 3.7 mg/dL) and demonstrating that choroidal perfusion impairment can be reversible. Indeed, a substantial proportion of organ damage in AAV is associated with delayed diagnosis and insufficient treatment, and early initiation of aggressive immunosuppression is critically important for preserving organ function. In our patient, effective and timely induction therapy, along with continuation of methylprednisolone at 1 mg/kg/day and administration of the second dose of cyclophosphamide following ocular involvement, led to rapid restoration of visual acuity to normal levels, which is noteworthy in this context [2, 3, 7].

The limited number of reported cases of choroidal involvement associated with MPA in the literature makes it difficult to draw definitive conclusions regarding its true frequency and clinical course. In a study evaluating 14 AAV patients followed for retinal vasculitis between 2006 and 2017, choroidal vasculitis was identified in 4 patients; however, all of these cases were reported to have GPA [11]. Nevertheless, existing reports emphasize that posterior segment involvement should not be overlooked, particularly in patients with severe systemic disease. In AAV patients who develop sudden visual loss or visual symptoms, anterior segment evaluation alone is insufficient; instead, appropriate imaging modalities capable of assessing choroidal circulation should also be utilized. This approach may facilitate diagnosis and positively influence visual prognosis through timely initiation of treatment [4–6, 12].

The present case contributes to the literature by demonstrating that choroidal ischemic involvement, although rare, may develop in MPA with severe renal disease and may be completely reversible with early diagnosis and appropriate immunosuppressive therapy. Furthermore, this case once again highlights that ocular findings in AAV should not be overlooked and underscores the importance of a multidisciplinary approach.

## Conclusion

Choroidal ischemia is an extremely rare manifestation in MPA and may lead to severe visual loss. The prognosis is poorer when it occurs together with renal involvement. A multidisciplinary approach and early aggressive immunosuppressive therapy are critically important for both renal and visual outcomes.

## Supplementary Information

Below is the link to the electronic supplementary material.

Supplementary Material 1

## Data Availability

All data generated or analysed during this study are included in this published article.
