# Supplementary material for: Reversible choroidal ischemia as a rare sight-threatening manifestation of microscopic polyangiitis presenting with crescentic glomerulonephritis
Source: BMC Nephrol. 2026 Apr 2;27:305. doi: 10.1186/s12882-026-04828-x (PMC13169638; doi:10.1186/s12882-026-04828-x)
Supplement: Supplementary file 1 — Supplementary Material 1 [file 12882_2026_4828_MOESM1_ESM.docx]

Supplementary Appendix 1. De-identified renal histopathology report summary

Specimen: Native kidney biopsy

Light microscopy

- Total glomeruli: 18
- Crescent formation: Present (14/18 glomeruli)
- Necrosis: Present (fibrinoid necrosis in several glomeruli)
- Global sclerosis: Present (2/18 glomeruli)
- Glomerular basement membrane thickness: Within normal limits
- Mesangial matrix/cellularity: Within normal limits
- Endocapillary hypercellularity/proliferation: Absent
- Segmental sclerosis: Absent
- Periglomerular fibrosis: Absent

Tubulointerstitial compartment

- Interstitial edema: Present
- Interstitial inflammation: Present (moderate mixed-type inflammatory cell infiltration)
- Tubulitis: Present (focal tubulitis)
- Tubular epithelial injury: Present (epithelial desquamation, loss of brush border, reactive atypia)
- Red blood cell casts: Present (in a few tubules)
- Calcification: Absent
- Tubular atrophy: Absent
- Interstitial fibrosis: Absent

Vessels

- Arteries/arterioles: No specific abnormality identified

Medulla

- No additional specific findings

Immunofluorescence

- Number of glomeruli examined: 4
- C3: 1+
- IgG: Negative
- IgA: Negative
- IgM: Negative
- C1q: Negative
- Kappa/Lambda: No evidence of monoclonality
  Interpretation: Overall findings are consistent with a pauci-immune pattern.

Electron microscopy

- Not performed / Not available

Final diagnosis

Necrotizing and crescentic glomerulonephritis
